# Supplementary figures and images for: Proteomics and Bioinformatics Identify Drug-Resistant-Related Genes with Prognostic Potential in Cholangiocarcinoma
Source: Biomolecules. 2024 Aug 8;14(8):969. doi: 10.3390/biom14080969 (PMC11352417; doi:10.3390/biom14080969)

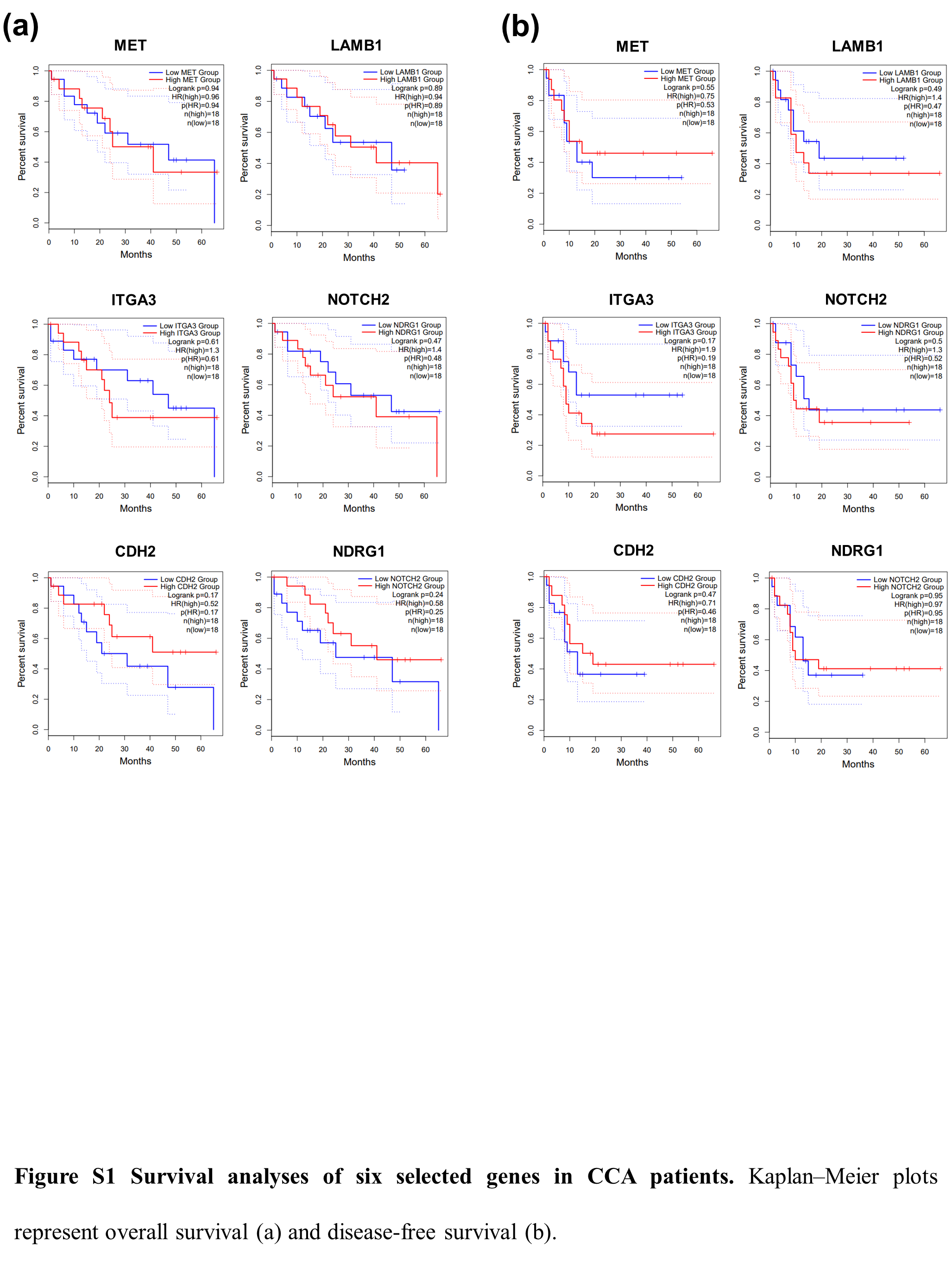

Supplement: Supplementary file 1 [file biomolecules-14-00969-s001.zip › FigS1.tif]

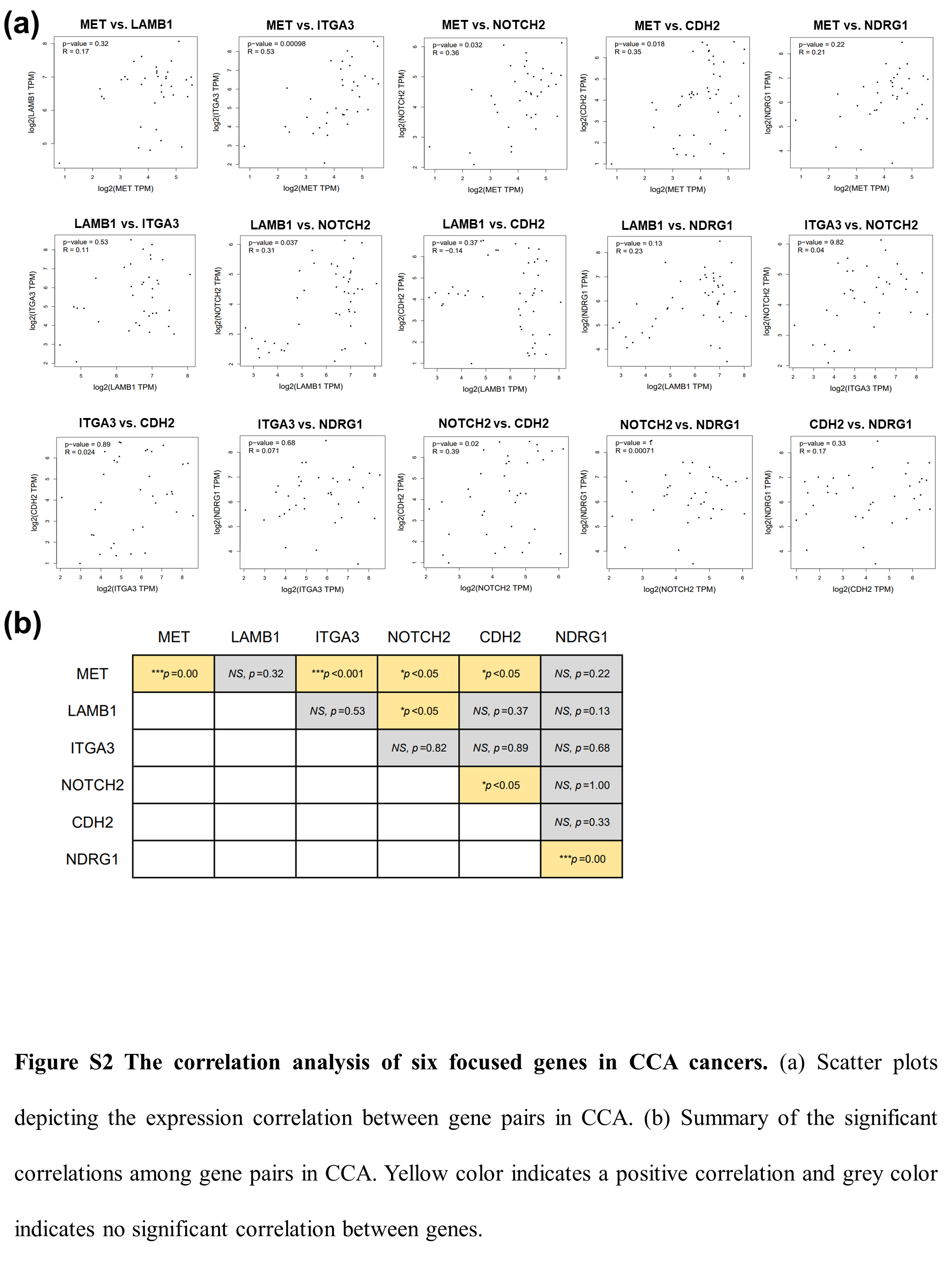

Supplement: Supplementary file 1 [file biomolecules-14-00969-s001.zip › FigS2.tif]
